# Quality of Life: Psychological Symptoms—Effects of a 2-Month Healthy Diet and Nutraceutical Intervention; A Randomized, Open-Label Intervention Trial (RISTOMED)

**DOI:** 10.3390/nu12030800

**Published:** 2020-03-18

**Authors:** Isabelle Bourdel-Marchasson, Rita Ostan, Sophie C Regueme, Alessandro Pinto, Florence Pryen, Zoubida Charrouf, Patrizia A d’Alessio, Claire Roubaud Baudron, Florent Guerville, Jessica Durrieu, Lorenzo M Donini, Claudio Franceschi, Luzia Valentini

**Affiliations:** 1 CRMSB UMR 5536, Université Bordeaux/CNRS, F−33000 Bordeaux, France; 2 CHU Bordeaux, Pôle de Gérontologie, Bordeaux, F−33000 Bordeaux, France; sophie.regueme@chu-bordeaux.fr (S.C.R.); claire.roubaud@chu-bordeaux.fr (C.R.B.); florent.guerville@chu-bordeaux.fr (F.G.); jessica.durrieu@chu-bordeaux.fr (J.D.); 3 Department of Experimental, Diagnostic and Specialty Medicine (DIMES), University of Bologna, 40126 Bologna, Italy; rita.ostan3@unibo.it (R.O.); claudio.franceschi@unibo.it (C.F.); 4 Experimental Medicine Department, Sapienza University of Rome, 00185 Rome, Italy; alessandro.pinto@uniroma1.it (A.P.); lorenzomaria.donini@uniroma1.it (L.M.D.); 5 Mendes SA, Via Giacometti 1, CH−6900 Lugano, Switzerland; fpryen@mendes-swiss.ch; 6 Department of Chemistry, University Mohammed V, Rabat BP 1014, Morocco; zcharrouf@yahoo.fr; 7 AISA (Anti-Inflammatory Senescence Actives) Therapeutics, Genopole Entreprises 91058 Evry, France; patriz.dalessio@gmail.com; 8 Université Bordeaux, INSERM UMR 1053, BaRITon, F−33000 Bordeaux, France; 9 Department of Applied Mathematics, Lobachevsky University, Nizhny Novgorod 603950, Russia; 10 Department Gastroenterology and Hepatology, Charité Universitätsmedizin Berlin, 10117 Berlin, Germany; valentini@hs-nb.de; 11 Neubrandenburg Institute of Evidence-Based Dietetics (NIED), University of Applied Sciences, 17033 Neubrandenburg, Germany

**Keywords:** inflammaging, depressive symptoms, nutraceuticals, quality of life

## Abstract

Depression symptoms and lower health-related quality of life (HRQoL) are associated with inflammation. This multicenter dietary intervention was shown to reduce inflammation in older people. This was the main outcome. Here, we describe the effects on HRQoL, anxiety, and depressive symptoms according to inflammation status. Overall, 125 healthy older subjects (65–80 year) were recruited (Italy, France, and Germany) and randomized into four arms (A, Healthy diet (HD); B, HD plus De Simone Formulation probiotic blend; C, HD plus AISA *d*-Limonene; D, HD plus Argan oil). The HD was weight maintaining, rich in antioxidant vitamins, polyphenols, polyunsaturated fatty acids (n6: n3 ratio = 3:1), and fiber. Data on inflammatory parameters, mental (MCS) and physical (PCS) component summaries of HRQoL (SF−36), anxiety symptoms (STAI state), and depressive symptoms (CES-D) were collected before and after 56 days of intervention. Body fat mass proportion (BFM) was considered a co-variable. A decrease of CES-D score was seen in the four arms (A: −40.0%, *p* = 0.001; B: −32.5%, *p* = 0.023; C: −42.8%, *p* = 0.004; and D: −33.3%, *p* = 0.21). Within the subgroups of subjects with medium/high inflammation a similar decrease in CES-D score occurred in all groups (A: −44.8%, *p* = 0.021; B, −46.7%, *p* = 0.024; C, −52.2%, *p* = 0.039; D, −43.8%, *p* = 0.037). The effect of interventions on CES-D was not related to baseline inflammation. MCS-HRQoL improved in A and C. There was no change in anxiety or PCS-HRQoL. In this trial with no control group, a decrease in depressive symptoms in healthy older volunteers was observed after a 2-month healthy diet intervention, independently of inflammation but with possible limitations due to participation.

## 1. Introduction

The inflammatory reaction includes, among other changes, behavioral changes such as apathy, lethargy, reduced locomotor activity, inhibition, anhedonia, anxiety, sleepiness, disinterest, hyperalgesia, and failure to concentrate. Together these symptoms constitute “sickness behavior” and are close to depressive symptoms [1,2]. These symptoms are of various intensities. Subclinical elevation of interleukin−6 (IL-6) and C-reactive protein (CRP) has also been associated with lower quality of life and depressive symptoms [3]. Another argument for the link between depressive symptomatology and low-grade inflammation is suggested by the improvement in sleep disorders observed with decreases in high-sensitivity CRP (hsCRP) levels in military personnel [4]. In obese animals, depressive symptoms were linked to cytokine levels secreted by adipose tissue or to chronic low-grade endotoxemia linked to increased gut permeability [5]. In all age groups, consistent evidence of association with levels of hsCRP and depressive symptoms [6,7] or impaired HRQoL (health-related quality of life) can be found [8]. Inflammation induces by humoral, neural, and cellular pathway changes in brain signaling, particularly brain function relevant to behavior (see [9] for review). The content of the diet was also shown to influence the level of depressive symptoms, with a high intake of carbohydrates associated with a higher level of depressive symptoms [10]. In an open-label randomized intervention trial with diet or exercise, weight loss induced a decrease in inflammation and improvement of HRQoL [11].

Inflammaging represents low-grade inflammation associated with senescence, increased vascular risk, and occurrence of age-related diseases [12]. The European project RISTOMED (www.ristomed.eu, CORDIS FP7) [13,14] was a multicenter, open-label, randomized controlled intervention trial (RCT) aimed at reducing the level of inflammation in older people by means of a healthy diet in association with the nutraceutical products VSL−3 probiotic blend [15], AISA *d*-Limonene [16], and argan oil [17]. It was conducted in a cohort of older and healthy non-obese non-malnourished subjects. The intervention with the healthy diet alone or with the nutraceuticals was associated with a decrease in inflammation level, particularly in those subjects with a medium-high inflammatory status at baseline and who received the AISA *d*-Limonene supplement [13]. The present study addresses a secondary objective of this RCT with no specific hypothesis for nutraceuticals effect on the outcomes, i.e., was to assess the effect of the dietary intervention on quality of life, depression, and anxiety. We aimed to describe the effect of the RISTOMED intervention on HRQoL, depressive symptoms, and anxiety in all participants and, particularly, in subjects belonging to the cluster with medium-high inflammatory status.

## 2. Materials and Methods

### 2.1. Participants

The RISTOMED trial has been previously described [13]. It was planned to include a population of 144 older volunteers in Italy, Germany, and France. Subjects were included if they were 65–85 year old, with a Body Mass Index (BMI) in the 22–30 kg/m^2^ range, functionally independent for basic daily living activities, and free of hematological, inflammatory, metabolic, hepatic, and renal diseases. They were excluded if they were affected by any neurodegenerative diseases, severe psychiatric diseases, or cardiovascular diseases or if they had pathological conditions or used medications or nutritional supplements that would interfere with one of the nutraceuticals. They should have been free from infections or antibiotics in the 20-day period before inclusion and were secondarily excluded in case of such events during the intervention period. The ethical committees in each country approved the protocol, which was recorded in ClinicalTrials.gov as NCT01069445–NCT01179789 and was executed under the 7th Framework Program of the European Commission.

### 2.2. Study Design

This was a multicenter international open-label randomized study with four parallel groups. The study was conducted according to the declaration of Helsinki (2008) and was approved by the ethics committees for Human Protection in Biomedical Research in France, Italy, and Germany. After receiving information about study procedures and signing an informed consent form, eligible volunteers were randomized into four arms: arm A: RISTOMED diet alone, arm B: RISTOMED diet + De Simone Formulation (DSF) probiotic blend, arm C: RISTOMED diet + AISA−5203-L orange peel extracted monoterpene *d*-Limonene, and arm D: RISTOMED diet + Native^®^Argan oil.

Volunteers were recruited in the community using advertising in newspapers, intranet hospital website, and word of mouth. The leaflet focused on access to the RISTOMED website, healthy diet, and provision of nutraceuticals. The information given to the participants mentioned that the aim of the main study was to improve or to maintain a healthy lifestyle. Expectations about decrease in inflammation, oxidative stress, or improvement in bowel comfort were also written but possible psychological benefits were not raised.

Randomization was performed centrally with stratification based on recruitment center, gender, and age categories (65–74 year and 75–85 year). A computer-generated randomization log was used for each stratum. It assigned the subject to his/her arm with equal probability according to the sequential number in the log. Volunteers were assessed at the baseline visit, after one month of intervention, and at the end of the two-month intervention. We present here the baseline and the two-month data.

### 2.3. Intervention

All subjects received a personalized diet intervention given through a web platform. The diet was designed by a dietitian according to current recommendations for older people and acknowledging concepts in clinical nutrition to decrease systemic inflammation and oxidative stress and optimize the gut microbiota. Recipes and daily menus were formulated, taking into account traditional and personal eating habits, assessed by the dietitian in an in-person nutritional assessment session at baseline. Foods known to be particularly rich in antioxidants (vitamin E, C, carotenoid, polyphenols), minerals (selenium, zinc), fiber (30 g/day, insoluble/soluble about 1:1), and polyunsaturated fatty acids (PUFA) were preferably chosen. PUFA aimed for the n6:n3 ratio = 3:1 based on clinical concepts to optimize the anti-inflammatory potential of n3-FA [18]. It was recommended to drink at least 1–1.5 mL water/kcal/day and to use a calcium-rich water (Ca 150 mg/L). The total energy intake was calculated from the Harris–Benedict equation multiplied by physical activity level coefficient for body weight stability. Full details of the intervention were previously reported [13].

Participants received daily menu suggestions electronically for one week in advance and could switch and swap menus to a certain extent for better integration into their daily life. All changes were controlled online by a dietitian and corrected if necessary. Weight was monitored weekly by self-reporting on the web platform and objectively controlled during the study visits at baseline and after 1 and 2 months. The diet was modified according to weight variations, if any occurred. Compliance with the intervention was also self-reported each day on the web platform. To avoid bias caused by increased physical activity, participants were encouraged to keep their usual activity pattern, and no specific instructions about physical activity were given by the study personnel.

The probiotic blend is the De Simone Formulation (DSM, supplied by ACTIAL Farmaceutica Lda and now available in Europe under the trademark Vivomixx^®^). It is formulated as a granulated powder of 112 billion lyophilized bacteria per capsule with defined ratios of *Streptococcus thermophilus* DSM 24731, bifidobacteria (*B. breve* DSM 24732, *B. longum* DSM 24736, *B. infantis* DSM 24737), and lactobacilli (*L. acidophilus* DSM 24735, *L. plantarum* DSM 24730, *L. paracasei* DSM 24733, *L. delbrueckii subsp. bulgaricus* DSM 24734). The product was administered orally, two capsules daily.

The monoterpene AISA 5203-L (*d*-Limonene and its metabolites) extracted from cold-pressed orange peel now available in Europe under the trademark AISA moleculum^®^ was administered orally in the form of soft gel capsules at the recommended dose of 10 ± 1 mg/kg body weight.

Native^®^Argan oil (Maison de Argan, Bordeaux, France), extra-virgin oil obtained using a cold-pressed technique from kernels contained in the *Argania spinosa* fruit, was supplied in 12.5 mL monodose sachets. The daily recommended intake was 25 mL taken as two monodoses per day at each person’s convenience, in substitution for a comparable amount of fat in the RISTOMED diet.

The intervention duration was two months.

### 2.4. Descriptive Variables

The baseline description of participants included age, gender, medical history, and concomitant medication. At baseline and at the end of the intervention, anthropometric measures, physical performance, physical activity, quality of life, anxiety, and depressive symptoms were assessed. Fasting blood sampling and stool collection were performed on the same occasions.

Anthropometric measures considered here are BMI (kg/m^2^), waist circumference (cm), and body fat proportion; we estimated the proportion of body fat mass based on skinfold values with the Durnin and Womersley equations for older people [19,20].

Independence for instrumental activities of daily living was assessed using the Lawton and Brody scale, with scores varying from 0 to 8, where 8 represents independence [21]. Handgrip strength was measured with a JAMAR© dynamometer, and the highest value of three attempts was recorded in kg for the dominant arm. The Short Physical Performance Battery (SPPB) tested balance (side-by-side stance, semi-tandem stance, and tandem stance), chair rise (strength), and gait speed over a 4 m distance. The maximal score is 12, corresponding to the highest performance.

Serum concentration of hsCRP (mg/L), erythrocyte sedimentation rate (ESR, mm/h), fibrinogen (mm/dL), Tumor Necrosis Factor-α (TNF-α, pg/mL), IL−6 (pg/mL), and white blood cell count were used to assess systemic inflammation; glucose metabolism was described with fasting blood glucose (mg/dL), fasting insulin (µU/m), and the insulin resistance assessment by the homeostatic assay: HOMA-IR = [glucose (nmol/L) * insulin (µU/mL)/22.5] [22]; lipid profile included total cholesterol and triglyceride concentrations (mg/dL).

### 2.5. Outcomes

The outcomes of the present study are changes in HRQoL, anxiety, and depressive symptoms according to the dietary interventions.

HRQoL was assessed using the general scale MOS SF−36 [23]. Items are aggregated into eight multi-item scales, called Physical Functioning (PF), Role-Physical (RP), Bodily Pain (BP), General Health (GH), Vitality (VT), Social Functioning (SF), Role-Emotional (RE), and Mental Health (MH). Items were transformed and summed according to the manual for the instrument to give scores from 0 to 100 for each scale, with a higher score indicating a better HRQoL. These eight scales were aggregated into two summary measures: the Physical (PCS) and Mental (MCS) Component Summary scores. MCS and PCS were recorded at baseline and after 2 months of dietary intervention.

The Center for Epidemiologic Studies Depression Scale (CES-D) 20-item scale was used to measure depressive symptomatology [24]. The higher the total score, the higher the level of depressive symptomatology expressed by the respondent; the possible total score ranges from 0 to 60. The State-Trait Anxiety Inventory (STAI) is an introspective psychological inventory consisting of 40 self-reported items pertaining to anxiety affect [25]. We considered the State Anxiety Score (STAI state). CES-D and STAI state were recorded at baseline and after one month and two months of dietary intervention.

### 2.6. Analysis

We assessed changes in descriptive variables and outcomes using the paired Wilcoxon test. Changes according to intervention arms A, B, C, and D are presented as percentages of the basal values.

We also explored relationships at baseline between waist circumference or body fat mass proportion and HRQoL, anxiety, depressive symptoms, and physical performance with partial correlation tests.

We have previously shown that the RISTOMED intervention has different effects according to the basal inflammation status [13]. As sensitivity analysis, we applied the same two-step cluster strategy based on baseline inflammatory markers described above. This two-step cluster analysis identified two clusters of subjects: “low inflammation” and “medium-high inflammation”. The changes in outcomes were analyzed according to membership in each cluster.

Two-month changes in outcomes according to treatment group controlled for age, gender, and fat proportion at baseline were analyzed with a general linear model for repeated measures. We explored the differences according to group in two steps: (1) all treatment groups, (2) effect of each treatment that included a nutraceutical (B, C, and D) compared with group A (diet only) if there was a treatment group effect.

## 3. Results

In the study, 138 subjects were recruited and randomized. Due to dropout and secondary exclusions for adverse events, 125 subjects (67 females, 70.4 years, SD 3.9) were considered in the analysis (Figure S1). The subjects’ baseline characteristics are presented in Table 1. Only two subjects were not fully independent according to IADL. Physical performance assessed with SPPB was close to the maximum. Eleven men and 26 women were dynapenic according to their handgrip strength [26]. None had a depression disease with CES-D score below the proposed threshold for diagnosis [24].

Compliance with the diet was 73.0% (SD 16.7); compliance with nutraceutical was between 87% and 95%, between 71% and 80%, and between 81% and 90% for VSL#3, AISA, and Argan Oil, respectively. No serious adverse event was related to the interventions (diet or nutraceuticals) [13].

As expected, we observed a positive correlation of body fat mass with female gender and a negative correlation of female gender or increased age with muscle strength at baseline. At baseline in the total population, body fat mass proportion was positively correlated with belonging to the medium-high inflammation cluster (r = 0.200, *p* = 0.031) and CES-D (r = 0.276, *p* = 0.002) and negatively correlated with MCS (−0.185, *p* = 0.041), but no correlation was seen for PCS (−0.224, *p* = 0.13) or STAI state (0.085, *p* = 0.351). These correlations were, however, weak. Female gender was associated with higher CES-D score (r = 0. 265, *p* = 0.003), higher STAIT state (r = 0.225, *p* = 0.011), and lower MCS (r = −0.251, *p* = 0.005). Baseline CES-D was not different in the low-inflammation cluster (7.7 ± 9.1) and the medium-high inflammation one (8.2 ± 6.8) (*p* = 0.136).

The global analysis of the two-month changes showed a decrease in ESR and a decrease of depressive symptoms according to CES-D in the four arms (Table 2 and Table 3). Within the subgroups of subjects with medium/high inflammation, we observed a similar decrease in CES-D score in all groups (A: −44.8%, *p* = 0.021; B, −46.7%, *p* = 0.024; C, −52.2%, *p* = 0.039; D, −43.8%, *p* = 0.037). However, the decrease of CES-D was similar in the medium-high baseline inflammation cluster as compared to the low inflammation cluster.

**Figure 1:**
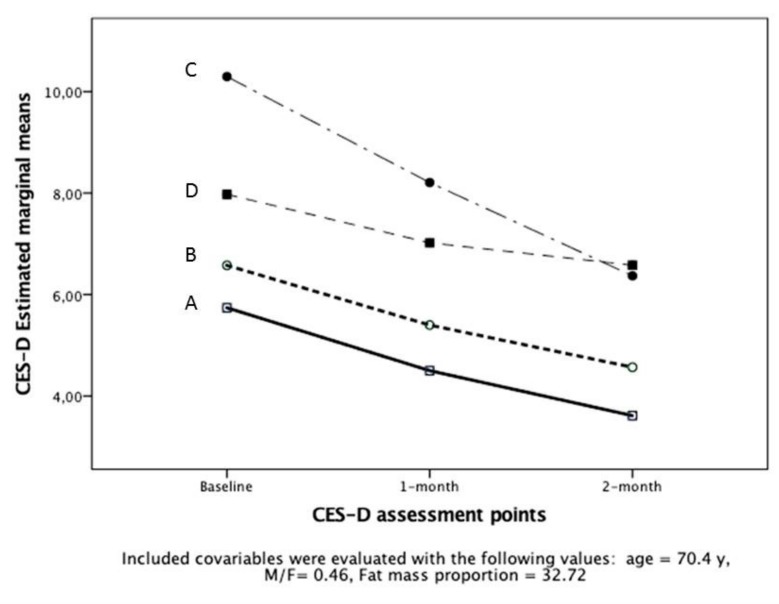
Changes in CES-D score according to time and intervention groups with adjustments for age, gender, and body fat proportion.

There was no treatment group effect on CES-D improvement (*p* = 0.098) (Figure 1).

MCS did not change over time in the total population, but there was an effect of treatment group (*p* = 0.034). The SF−36 MCS increased in arms A (*p* = 0.020) and C (*p* = 0.025). We performed a post hoc analysis to investigate the possible reasons for this treatment group effect. We compared baseline and end of intervention MCS across groups. Despite randomization we observed a lower mean baseline MCS in group C as compared with group A (*t* test, *p* = 0.048, mean difference 4.14, 95% confidence interval of the difference 0.46–8.22), with group B (*t* test, *p* = 0.035, mean difference 4.15, 95% confidence interval of the difference 0.31-8.00). The end-of-intervention MCS was similar across groups.

No changes were observed in SF−36 PCS. There were no effects of interventions on STAI state, handgrip strength, or SPPB and no effect according to treatment group for each.

## 4. Discussion

Depressive symptoms of healthy older subjects decreased after a two-month healthy diet adapted to three different European countries’ populations, independently of nutraceutical intake. The decrease appeared to be continuous with time, with an intermediate value at one month. There was no consistent change in any of the other clinical variables. This healthy diet and the nutraceutical products were given with the aim of decreasing systemic inflammation and oxidative stress and optimizing gut microbiota, as reported previously [13].

The depressive symptoms observed here failed to achieve a diagnosis of depression since the level of the symptoms was below the lower threshold used for psychiatric diagnosis (16/60) [24]. The range of CES-D values we observed was similar to that of a young adult population included in a large longitudinal study [27]. In these young subjects, cumulative exposure to such a level of depressive symptoms was shown to be associated with increased incidence of vascular wall pathology. We thus estimated a clinically meaningful level of such symptoms. Baseline body adiposity was related to CES-D symptom intensity at baseline in our study. A decrease in fat mass could have induced a decrease in CES-D score. However, we gave a weight-maintaining diet and did not propose any physical activity program or provide advice on physical activity. A small decrease in BMI was observed, but we did not find any significant change in body composition. Therefore, improvement in CES-D in this study cannot be attributed to a change in adiposity. We expected to find a higher decrease in CES-D in the cluster with the higher level of inflammation, but we did not. In this short period (2 months), few other mechanisms may explain the improvement. Several nutrients intake may improve with this healthy diet and played a role in the decrease of depressive symptoms. A prospective cohort observed that the risk of depression was increased with more than four nutrients not meeting the recommended intake [28]. The healthy diet of the present study was in line with recommendations for depression prevention [29].

Better quality in diet was shown associated with lower incidence of depression according to a meta-analysis [30]. Feeding behavior is, however, likely associated with mood, cognition, education, and socioeconomic status; each of these items are confounders to take into account. Randomized controlled trials (RCT) are therefore necessary to draw conclusions regarding the protective or curative effect of healthy diet on depressive symptoms. In subjects with spinal cord injury, an open-label controlled study concluded that an anti-inflammatory diet decreased depressive symptoms [26]. These subjects had a higher intensity of depressive symptoms, with baseline CES-D means of 14.5 and a decrease of almost 50%. This was an exploratory study with no sample size calculation. In subjects with metabolic syndrome, a weight-decreasing diet intervention was shown to be associated with simultaneous decreases in adiposity, inflammatory markers, and depressive symptoms [27]. An RCT in major depression in adults showed an improvement in the dietary intervention group [31]. Diet was built according to recommendations for depression prevention [29]. The decrease in depressive symptoms in our healthy older population may be attributable to the diet itself.

The decrease in CES-D symptoms may also be due to participation in the study itself. Indeed, participation as a volunteer may have been a positive experience. The Hawthorne effect describes the improvement of behavior due to the fact that subjects are observed [32]. The exact impact of this effect on the study results is difficult to quantify. However, mental HRQoL or anxiety did not improve or slightly improved with the intervention. The level of anxiety symptoms assessed by STAI state at baseline was in the range of normative values given in people of similar age [25]. These symptoms were not related to adiposity at baseline and did not change with the interventions. We observed, in contrast, important and consistent changes in depressive symptoms in the four groups.

This study has some limitations. Despite randomization, intervention groups may differ on several characteristics, and we did not adjust comparisons between groups on potential confounding factors. The dietary intervention was short and significant changes in body composition could not be assessed. There was no control group without nutritional intervention for comparison and physical activity was not monitored. The expectation bias probably exists, because we recruited volunteers; the outcomes were subjective, i.e., psychological symptoms. Despite the fact that no information given during recruitment and inclusion addressed any psychological benefit, it is not possible to exclude that the result on depressive symptoms was due to the participation to the trial. The number of subjects to include was estimated based on expected hsCRP decrease and not based on decrease in depressive symptomatology. It is thus possible that the study was underpowered to distinguish the effects of each nutraceutical as an addition to the diet on depressive symptoms. In these healthy subjects, the margin of improvement may be very narrow due to the mild level of baseline depressive symptoms, and we cannot conclude that there was a lack of effect of the supplements. Finally, we have no full explanation about the mechanism of the origin of improvement in depressive symptoms.

This study has some strengths. This is a cross-cultural study with diets adapted to each country, and applicability is consequently high. The study also benefits from a comprehensive set of biological assessments of inflammation that evidenced an improvement after intervention [13]. Finally, this is the first interventional study exploring the effects of nutritional intervention on depressive symptoms in healthy older people.

## 5. Conclusions

In this older population, depressive symptoms improved with a healthy diet intervention that aimed to decrease inflammation and oxidative stress and improve the microbiota. However, mechanisms leading to this improvement were not evidenced in the present study, which included only healthy, non-obese, and non-malnourished older subjects. An RCT with a control group testing the effect of this healthy diet in older subjects with depressive symptoms as the main outcome is necessary to confirm these results.

## Figures and Tables

**Table 1:** Participants’ baseline characteristics according to their allocation groups.

Mean (SD)	Arm A Healthy Diet (HD) *n* = 31		Arm B HD + De Simone Formulation (Probiotics) *n* = 31		Arm C HD + Monoterpene AISA 5203-L *n* = 30		Arm D HD + Native^®^Argan Oil *n* = 33	
Age (years)	70.5	(4)	69.7	(3.9)	70.0	(3.8)	71.4	(3.9)
Gender: women, n (%)	17	(50)	16	(50)	16	(50)	18	(50)
BMI (kg/m^2^)	26.9	(3.4)	26.7	(3.8)	26,8	(2,9)	26.7	(3.5)
Waist circumference (cm)	90.6	(15.7)	94.5	(12.9)	94.1	(9.9)	92.8	(13.5)
Fat mass proportion (% body)	32.4	(7.1)	32.4	(6.1)	33.7	(6.4)	32.9	(6.3)
Function								
SPPB (0–12)	11.2	(1.8)	11.3	(1.2)	11.7	(0.7)	11.3	(1.2)
IADL (0–8)	8.0	(0)	7.8	(0.6)	8.0	(0)	8.0	(0)
Hand-grip strength (kg)								
Women	20.4	(4.9)	21.3	(3.7)	21.3	(4.9)	22.2	(7.5)
Men	38.0	(10.7)	39.1	(8.4)	39.1	(12.5)	37.9	(8.3)
MOS SF−36 (0–100)								
MCS	53.5	(7.6)	53.6	(6.6)	49.4	(8.3)	52.4	(6.5)
PCS	54.6	(9.9)	55.0	(8.4)	56.4	(6.7)	54.8	(6.8)
CES-D (0–60)	6.7	(6.7)	6.7	(5.4)	10.7	(10.2)	8.1	(7.7)
STAI state (20–80)	35.7	(11.4)	35.6	(10.1)	38.2	(11.6)	38.4	(10.1)
Fasting glucose (mg/dL)	94.5	(23.0)	92.5	(12.6)	95.2	(13.2)	93.7	(17.3)
Insulin (µU/mL)	9.0	(6.2)	9.0	(7.2)	10.6	(9.4)	10.2	(6.8)
HOMA-IR index	2.3	(1.9)	2.2	(2.1)	2.2	(1.1)	2.6	(2.4)
Cholesterol (mg/dL)	215	(42)	227	(44)	229	(43)	224	(38)
Triglycerides (mg/dL)	120	(64)	112	(48)	110	(54)	109	(48)
WBC (10^3^/mmc)	6.07	(1.41)	5.97	(1.47)	5.92	(1.14)	6.21	(1.48)
hsCRP (mg/L)	3.6	(3.6)	2.9	(3.8)	3.6	(4.9)	2.5	(2.6)
ESR (mm/h)	24.4	(18.2)	23.5	(21.2)	20.9	(13.6)	21.2	(15.7)
Fibrinogen (mm/dL)	377	(96)	394	(104)	379	(105)	359	(92)
TNF-a (pg/mL)	60.4	(151.7)	7.2	(17.9)	8.3	(27.7)	15.3	(53.6)
IL−6 (pg/mL)	39.9	(73.0)	12.3	(10.0)	30.1	(67.4)	18.9	(23.5)

**Table 2:** Percentage of median change from baseline for anthropometric measures, lipid profile, insulin resistance markers, and inflammatory profile for each dietary arm.

Changes from Baseline	ARM A Healthy Diet (HD) *n* = 31	*p*	ARM B HD + De Simone Formulation (Probiotics) *n* = 31	*p*	ARM C HD + Monoterpene AISA 5203-L *n* = 30	*p*	ARM D HD + Native^®^ Argan Oil *n* = 33	*p*
**Anthropometric measures**								
BMI (kg/m^2^)	−1.1%	0.005	−0.7%	0.012	−0.2%	0.028	0.0%	0.400
Waist circumference (cm)	−0.9%	0.112	0.0%	0.190	0.0%	0.449	0.0%	0.602
Body fat mass proportion (%)	+0.4%	0.295	+0.4%	0.223	−0.1%	0.667	+0.4%	0.190
**Lipid profile**								
Total cholesterol (mg/dL)	−2.7%	0.002	−3.4%	0.120	+1.7%	0.936	−1.75%	0.401
Triglycerides (mg/dL)	−5.6%	0.383	−2.7%	0.739	+10.0%	0.218	−4.6%	0.372
**Insulin resistance markers**								
Glucose (mg/dL)	−2.1%	0.029	0.0%	0.682	−4.3%	0.005	−0.9%	0.147
Insulin (microU/mL)	−1.0%	0.264	−2.8%	0.111	−20.4%	0.039	−1.7%	0.597
HOMA-IR Index	−17.1%	0.126	−7.9%	0.262	−23.1%	0.023	−2.9%	0.526
**Inflammatory profile**								
WBC (10^3^/mmc)	−2.5%	0.347	0.5%	0.313	−1.9%	0.210	−1.8%	0.191
hsCRP (mg/L)	−7.7%	0.688	0.0%	0.712	−7.1%	0.313	0.0%	0.757
ESR (mm/hr)	−32.8%	0.023	−28.6%	0.046	−46.5%	0.021	−35.3%	0.011
Fibrinogen (mg/dL)	+6.4%	0.456	−1.4%	0.532	−7.0%	0.082	+2.9%	0.137
IL−6 (pg/mL)	+18.2%	0.127	+15.9%	0.073	−16.1%	0.217	−3.7%	0.636
TNF-α (pg/mL)	0.0%	0.322	0.0%	0.225	0.0%	0.765	0.0%	0.614

BMI (Body Mass Index); HOMA-IR (Homeostatic Assay for Insulin-Resistance); WBC (white blood cell count); hsCRP(high-sensitivity CRP); ESR (erythrocyte sedimentation rate); TNF-α (Tumor Necrosis Factor). Changes between baseline and end of intervention were analyzed with paired Wilcoxon test.

**Table 3:** Percentage of median change from baseline in physical, depression and anxiety tests for each dietary arm.

Changes from Baseline	ARM A Healthy Diet (HD) *n* = 31	*p*	ARM B HD + De Simone Formulation (Probiotics) *n* = 31	*p*	ARM C HD + monoterpene AISA 5203-L *n* = 30	*p*	ARM D HD + Native^®^ Argan oil *n* = 33	*p*
SPPB (0–12)	0.0%	0.856	0.0%	0.297	0.0%	0.538	0.0%	0.672
Handgrip Strength Test (kg)	−0.7%	0.289	−1.7%	0.175	−3.6%	0.104	0.0%	0.999
MOS SF36-PCS (0–100)	1.6%	0.277	1.6%	0.524	−1.1%	0.905	0.0%	0.727
MOS SF36-MCS (0–100)	2.0%	0.020	0.8%	0.938	2.1%	0.025	−0.3%	0.446
CES-D (0–60)	−40.0%	0.001	−32.5%	0.023	−42.8%	0.004	−33.3%	0.021
STAI state (20–80)	0.0%	0.675	0.0%	0.798	−2.3%	0.375	0.0%	0.468

SPPB (Short Physical Performance Battery); MOS SF−36 MCS (Mental Component Summary) and PCS (Physical Component Summary); CES-D (Center for Epidemiologic Studies Depression Scale); STAI state (State-Trait Anxiety Inventory, State score). Changes between baseline and end of intervention were analyzed with paired Wilcoxon test.

## Supplementary Materials

The following are available online at https://www.mdpi.com/2072-6643/12/3/800/s1, Figure S1: Consort flow diagram of the RISTOMED study.
