# Supplementary figures and images for: Quality of Life: Psychological Symptoms—Effects of a 2-Month Healthy Diet and Nutraceutical Intervention; A Randomized, Open-Label Intervention Trial (RISTOMED)
Source: Nutrients. 2020 Mar 18;12(3):800. doi: 10.3390/nu12030800 (PMC7146172; doi:10.3390/nu12030800)

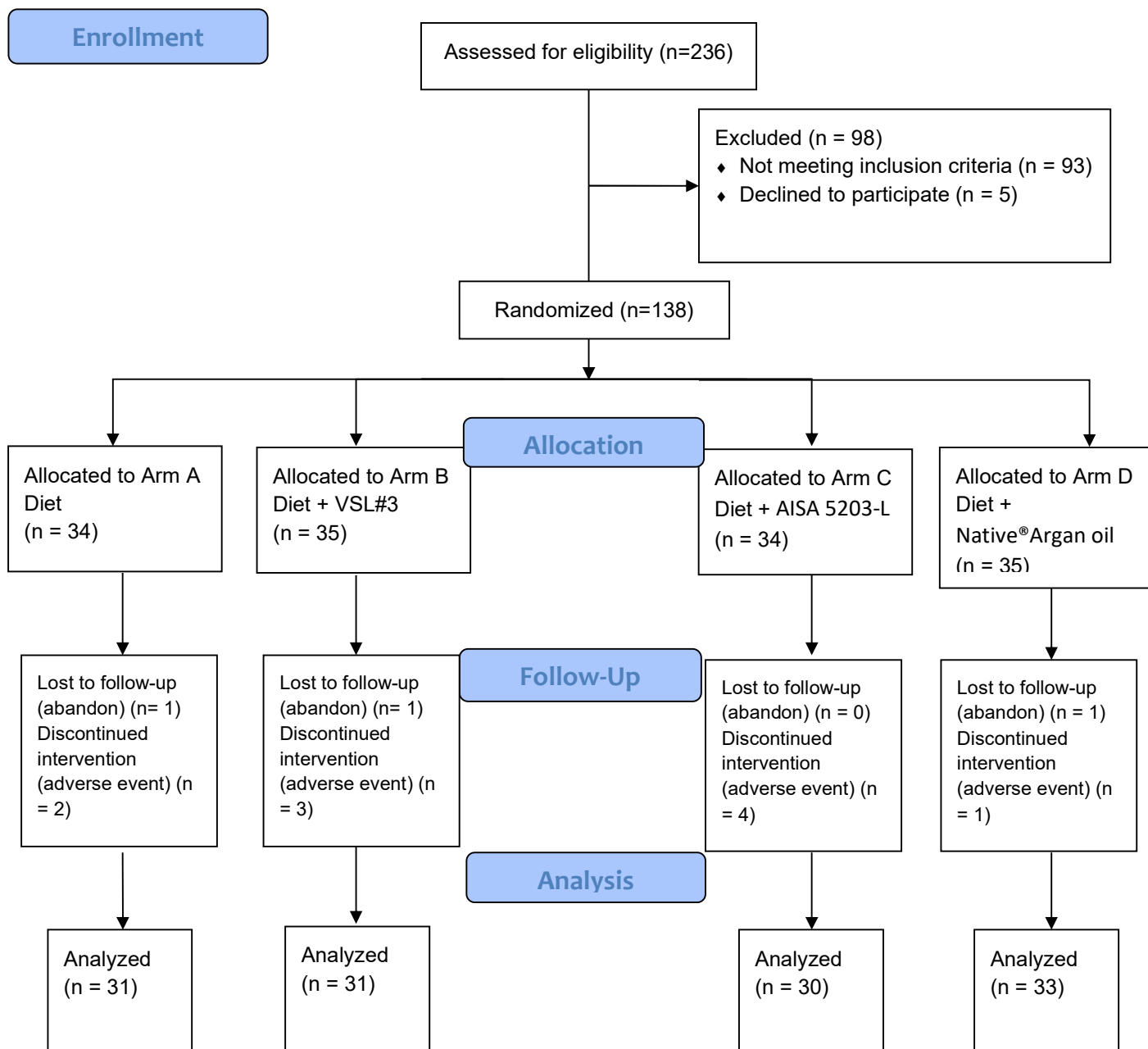

Supplement: Supplementary file 1 [file nutrients-12-00800-s001.pdf]
